# Access to Dental Care—A Survey from Dentists, People with Disabilities and Caregivers

**DOI:** 10.3390/ijerph18041556

**Published:** 2021-02-06

**Authors:** Gianmaria D’Addazio, Manlio Santilli, Bruna Sinjari, Edit Xhajanka, Imena Rexhepi, Rocco Mangifesta, Sergio Caputi

**Affiliations:** 1 Department of Innovative Technologies in Medicine and Dentistry, University “G. d’Annunzio” of Chieti-Pescara, 66100 Chieti, Italy; gianmariad@gmail.com (G.D.); santilliman@gmail.com (M.S.); imena.rexhepi@unich.it (I.R.); scaputi@unich.it (S.C.); 2 Department of Dental Medicine, Medical University of Tirana, Rruga e Dibrës, 1001 Tirana, Albania; edit.xhajanka@umed.edu.al; 3 Occupational Allergy, Nanomaterials and Fibers Biosafety and Immunotoxicology Group, CAST, University “G d’Annunzio” of Chieti-Pescara, 66100 Chieti, Italy; r.mangifesta@unich.it

**Keywords:** special care, oral health, health disparities, disabilities, special needs

## Abstract

The literature highlights differences in the dental conditions of people with disabilities compared with the general population. The aim of this study was to provide an overview of the dental health of people with disabilities in order to understand if their needs are met and to identify their most critical issues as per dentists. A paper and a Google Form platform were used in conducting a survey in Central Italy (the Abruzzo region), by performing an analysis on different points of view as reported by people with disabilities and dentists. The results showed that only 69.2% of dentists treat persons with disabilities. Of these, 73.5% treat less than 10 patients with physical disabilities per year. However, 54% of dentists do not treat people with cognitive impairment and a poor ability to collaborate during treatment. More than 80% of respondent dentists report that people with disabilities do not have good oral hygiene. On the other hand, 49.1% of people with disabilities (or their caregivers in cases where the patient was unable to answer) report that they rarely or never go to the dental office. Moreover, when they do go, it is mainly for emergencies. Despite this, respondents are well aware of their dental problems. However, they have difficulties in communicating their dental problems to their dentist. The 50% of dentists who treat people with cognitive impairment do not include them in follow-up, while only 20% of these patients reported being regularly recalled. This illustrates the importance of the implementation of follow-up. In addition, training courses could help clinicians to reduce this gap and create barrier-free dental offices.

## 1. Introduction

To date, it is estimated that there are nearly 3.1 million people with disabilities in Italy living with families, which corresponds to approximately 5% of the overall population. The United Nations Convention in 2006 defined people with disabilities as “those who have long-term physical, mental, intellectual or sensory impairments which in interaction with various barriers may hinder their full and effective participation in society on an equal basis with others” [1]. Of the 3.1 million people, 26.9% live alone, 26.2% live with their partner and 17.3% live with both their partner and children. Moreover, 7.4% live with their children and without their partner, and about 10% live with one or both parents; the remaining 12% live in other types of households [2]. Almost 1.5 million people aged seventy-five years or older have a disability, and 990,000 of them are women [2]. It has been reported that having a disability increases the likelihood of invasive and degenerative diseases in the elderly, leading to an increase in the number of people needing healthcare [3,4,5]. The assistance and care of people with disabilities by family members, caregivers and specialty facilities should guarantee a person’s total well-being [3]. In some cases, however, some health or social problems are actually considered secondary for a variety of reasons. Among these, access to dental care is difficult [6,7]. In this regard, international literature is often very discordant. Some studies mention that between 35% and 80% of people with disabilities have no difficulty in accessing dental care [8,9,10], while other studies have found that between 50% and 70% of people with disabilities have had negative experiences as they faced some level of difficulty in receiving dental care [11,12]. There are several reasons for this discrepancy, namely, proximity to dental offices, presence or absence of physical features that limit or prevent people with disabilities from obtaining services that are offered, lack of knowledge of the problem by the family members, attitudes of the dental staff, and type of disability [11,12,13]. In addition, difficulties in accessing care have also been described in other aspects of general medicine, very often due to problems related to transport [14]. However, the literature clearly shows that people with disabilities have worse overall dental conditions compared to the general population with the same age and social status [7,15]. In this sense, periodontal pathologies and orthodontic conditions were more present in people with disabilities. The same applies to caries indexes, although the literature does not provide clear evidence in this case [16,17,18]. In any case, about 38% of the total Italian population go to the dentist at least once a year. Economic issues are the main reason why people did not have dental visits [19].

It has also been widely demonstrated how some disabilities can be associated with concomitant oral pathologies such as oral respiration, macroglossia or recurrent traumatism [18,20], thus leading to a consequent increase in the incidence of pathologies affecting teeth and gums [6]. Furthermore, especially when addressing the needs of people with cognitive impairment, the difficulty to communicate pain or dental problems should be considered with the highest level of attention. The lack of knowledge of the problem, motor inability, frequent presence of dental misalignment or malocclusion and stagnation of oral fluids may be often associated with decreased oral hygiene standards [21].

Many efforts have been made over time to provide indications for the improvement of dental care skills by the dental services present in Italy. In this regard, in 2017 [22] and in early 2019 [23], the Italian Ministry of Health provided guidelines on “Indications for taking care of the patient with special needs who needs odontostomatological care” (directly translated as “Indicazioni per la presa in carico del paziente con bisogni speciali che necessita di cure odontostomatologiche”). These guidelines define the patient with disabilities as “the one who, in preventive, diagnostic and therapeutic operations, requires different times and methods than the standard ones”. In order to provide the most suitable dental care to people with disabilities, a classification system based on the degree of autonomy and ability to collaborate was, therefore, proposed in the definition of people with disabilities [23] as follows: collaborating and autonomous patients (meaning “Pazienti collaboranti e autonomi”), poorly collaborating and autonomous patients (meaning “Pazienti scarsamente collaboranti e autonomi”) and non-autonomous but collaborating or poorly collaborating patients and uncooperative patients (meaning “Pazienti non autonomi, ma collaboranti o scarsamente collaboranti e non collaboranti”). Moreover, the Italian guidelines also define a “non-collaborative patient” as someone who, due to health fragility and vulnerability or psychic or sensory disability, is unable to collaborate in dental treatment. These patients require a properly equipped operating environment and highly trained medical and care staff [22,23]. Furthermore, dentistry services are active in Italy in public hospitals that allow people with disabilities to obtain dental care in a hospital. Following this classification, the way in which patients are cared for in hospitals and the therapeutic approach have been also described in the guidelines. To the best of the authors’ knowledge, there are no data available which describe the environment of offered medical and dental assistance to people with disabilities in Central Italy.

The aim of this study was to provide an overview of the dental health of people with disabilities as reported both by the people with disabilities and the dentists and identify the most critical issues encountered.

The null hypothesis was that no differences and difficulties were present from both people with disabilities’ and dentists’ perspectives.

## 2. Materials and Methods

### 2.1. Study Design and Sample Selection

This survey was conducted in Abruzzo, in the Central Italy region, between December 2018 and April 2019, through an anonymous paper survey and a Google Form platform survey.

The subjects were selected from specific groups, in order to obtain a good stratification of the sample, a stricter control and a potentially higher responsiveness rate. Membership of category associations was the inclusion criterion. In particular, the groups analyzed in the present study were the following:

- Dentists working in public and private offices located in the provinces of Chieti and Pescara, in the Abruzzo Region, and members of the “National Association of Italian Dentists” (ANDI- Associazione Nazionale Dentisti Italiani);
- People with disabilities enrolled in the “National Association of Families of People with Intellectual and/or Relational Disabilities in Abruzzo” (ANFFAS—Associazione Nazionale Famiglie di Persone con Disabilità Intellettiva e/o Relazionale) or the family members and caregivers of people with disabilities who could not complete the questionnaires by themselves.

Two different questionnaires, one for each group, were prepared.

The optimization of the detection tools started from the analysis of questionnaires in the literature [13,24], then moving forward with some interviews to ANFFAS operators and family members or caregivers as well as to dentists.

Subsequently, the questionnaires prepared were subject to specific pre-tests, conducted on a selected group of people, in order to test the detection tools’ validity and reliability.

The pre-test was conducted with fifteen subjects, representative of the situation being investigated: The Regional President of ANFFAS; two operators of the ANFFAS Pescara division; four people with disabilities or family members from the ANFFAS association; and eight dentists belonging to the Dental Clinic, Department of Medical, Oral and Biotechnological Sciences, “Gabriele d’Annunzio” University, Chieti-Pescara and the “F. Renzetti” hospital, Lanciano (Chieti). The pre-test was useful to reduce bias and to obtain an accurate statistical analysis. On the basis of the feedback of the people taking part in the pre-test, authors modified the construction of the questions in order to make them more specific and to eliminate some. With the support of the ANFFAS, the questionnaires were translated into easy-to-read language, to make the questions easily understandable to people with disabilities. Then, they were administered to these people and to their caregivers through the ANFFAS Regional association. The same people that performed the pre-test were included in the analysis, before the full administration. In this way, the Cohen’s kappa coefficient, useful for the validity, reliability and consistency of the detection tools was calculated.

A sample-size calculation was conducted for a power of 80%, with a sampling error set at 5% using the probability proportional to the population size method. Based on a previous published study, a total of 85 dentists and 50 people with disabilities were required for data collection [11]. The questionnaire was sent to 550 ANDI members.

### 2.2. Questionnaires

The methodology adopted for the creation of the questionnaires allowed us to use quantitative variables that were differently distributed.

The questionnaires were delivered both on paper and via the Google Forms platform. The latter is a tool offered by Google which provides all information collected through customized surveys, as rendered in a spreadsheet including all answers to the survey in an anonymous way. All the questionnaires and the privacy approval form are included as Supplementary Materials.

#### 2.2.1. Questionnaires for Dentists

The questionnaire addressed to the dentists consists of three distinct sections. The first investigated a series of independent variables relating to age, sex, place of work and educational background. The second section included a set of questions related to dental issues and was divided into three subgroups based on the type of disability: physical, collaborating cognitive disability and non-collaborating cognitive disability. Specifically, as already described, the sub-categories were proposed following the classification of the Italian Ministry of Health [23]. This classification was adapted to the dental needs following the administration of the pre-tests. It was reduced to three categories:

- Physical disability, including people with pathologies that may require special management and relationship skills or the need to eliminate physical barriers to the treatment itself;
- Collaborating people with cognitive disabilities (people who, due to the fragility and health vulnerability or psychic or sensory disability, have lost the ability to take care of their own oral health);
- Non-collaborating people with cognitive disabilities (who are unable to collaborate in dental treatments, with severe psychic or sensory disabilities).

The last section of questions aimed to improve the knowledge regarding the approach, the achievable levels of care and training opportunities.

#### 2.2.2. Questionnaires for People with Disabilities

The questionnaire for people with disabilities, composed of 28 questions, initially investigated a series of independent variables relating to age, gender, city or residence province. Then, a set of questions was asked to understand the frequency of visits to the dentist, the types of treatments received, the barriers they experienced, the main difficulties encountered during treatments, the habits related to oral hygiene and whether it was in a dedicated facility or a private practice. The same questionnaire was then translated by some professional trainers belonging to the national ANFFAS, into easy-to-read language, aiming to make the questions more understandable and accessible to people with cognitive impairment.

If the patient was unable to answer the questions, the questionnaire was submitted to their caregiver or their family member. The results were presented together.

### 2.3. Statistical Analysis

The processing and analysis of data were carried out through statistical methodologies relating to the distribution of frequency concerning the unvaried data analysis. Some of the answers were codified as dichotomous variables, namely, as yes/no responses, whilst when a multiple choice selection was required, they were codified as categorical variables. Given the nature of our survey, we computed descriptive statistics for most of the questions. For each question, we computed the percentage of respondents that gave a particular answer with respect to the number of total responses to the question. In addition to descriptive statistics, other statistical methods were applied to investigate possible relationships between variables. The χ^2^ test was used to determine whether there is a statistically significant difference between the expected frequencies and the observed frequencies in some categories. Categories investigated were the following: difference between the specialist’s gender and attitude in treating patients with disabilities; oral hygiene status in the different category of disability analyzed; the difficulty expressed by dentist to understand the needs of people with disabilities; use of special equipment to perform dental treatment (wheelchair, special maintenance support and sedation).

All statistical comparisons were conducted with a significance level of *p* < 0.05. Statistical analyses were performed using the GraphPad version 8 (GraphPad Software 2365 Northsides Dr. Suite 560 San Diego, CA 92108, USA) statistical software.

### 2.4. Ethical Consideration

Participants received an information sheet and provided their informed consent in accordance with the EU General Data Protection Regulation GDPR (UE) n. 2016/679 before beginning the survey. Data collection took place between December 2018 and April 2019. The study protocol was post-approved by the Ethical Committee of the University of Medicine of Tirana on 15 January 2021. The survey was submitted with full anonymity. Questions were structured in a manner so that it was not possible to recognize responders’ identities from the answers provided. Additionally, respondents were informed of the nature of the study and decided to freely take part in it. The privacy approval form and all information regarding the study are included as Supplementary Materials.

## 3. Results

### 3.1. Results from Dentists’ Questionnaire

Table 1 shows the demographic data and the results obtained from the questionnaires administrated to the dentists. After administering the questionnaires, data relating to 91 participating subjects emerged. Given the total number of members of the two sections (550 in total), the response rate was around 16.5%. Out of these, 55 participants (60.4%) were male within an age range between 25 and 69 years. More than 35% of the total were aged between 28 and 30 years. Meanwhile, 81 (89.5%) dentists that participated had a degree in dentistry, and 56 (61.5%) did not hold a specialization degree.

**Figure 1:**
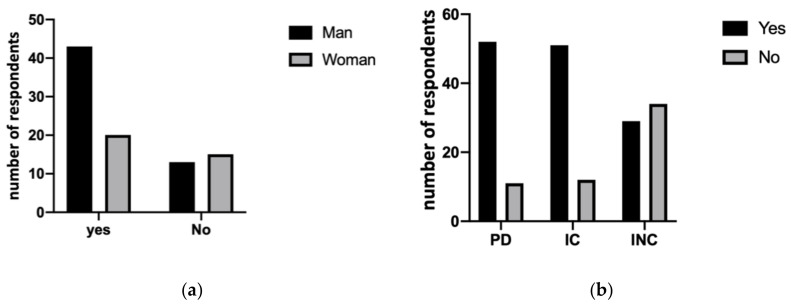
(**a**) Results of χ^2^ test between males and females showed no statistically significant difference in terms of treating (yes) or not treating (no) people with disabilities (*p* = 0.0628). (**b**) Evaluation of differences between physical disability (PD), collaborating (IC) and non-collaborating (INC) intellectual disabilities between those who reported treating (yes) or not treating (no) patients (*p* < 0.001).

No statistically significant difference was shown between dentists’ genders in terms of treating or not treating people with disabilities (*p* = 0.0628). As shown in Figure 1a, 49 dentists (54%) declared that they do not treat non-collaborating people with cognitive disabilities. A statistically significant difference emerged between those who reported treating or not treating people with physical disabilities, people with intellectual impairment but collaborating and people with intellectual impairment and non-collaborating (*p* < 0.001), as shown in Figure 1b.

Among the dentists who treat people with physical disabilities, 73.5% said that they treat less than 10 patients per year. Similarly, 76.5% treat less than 10 people with cognitive and collaborative disabilities and 93% less than 10 non-collaborative people with cognitive disabilities.

In total, 32.7% of dentists declared providing specific training for their dental team. The percentage rises to 33.3% in cognitive and collaborating people with disabilities and 41.4% in non-collaborating people but without statistically significant differences between groups (*p* = 0.70).

Eighteen dentists (34.6%) are disposed to make home visits for people with physical disabilities, in addition to 20 (39.2%) for collaborating people with cognitive impairment and 13 (44.8%) for non-collaborating people with cognitive disabilities.

**Figure 2:**
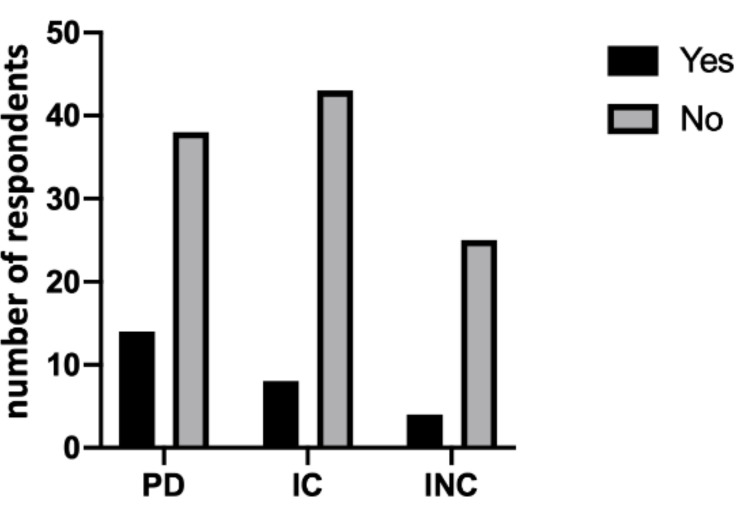
Oral hygiene differences (yes/no) between the three groups. No statistically significant differences were shown (*p* = 0.23).

Regarding dental treatments, 73.1% (38) of dentists believe that people with physical disabilities do not have good oral hygiene. This percentage worsens in people with collaborating (84.3%, 43) and non-collaborating cognitive disabilities (86.2%, 25). However, no statistically significant differences were found between the three groups in terms of good standard oral hygiene (*p* = 0.23), as shown in Figure 2. This condition is attributed, for people with physical disabilities, to the lack of dedicated facilities (52.5%) and to the physical accessibility (77.5%). On the contrary, 84.8% of collaborating people with cognitive disabilities and 81.5% of non-collaborating people believe that there is a lack of attention to oral hygiene.

**Figure 3:**
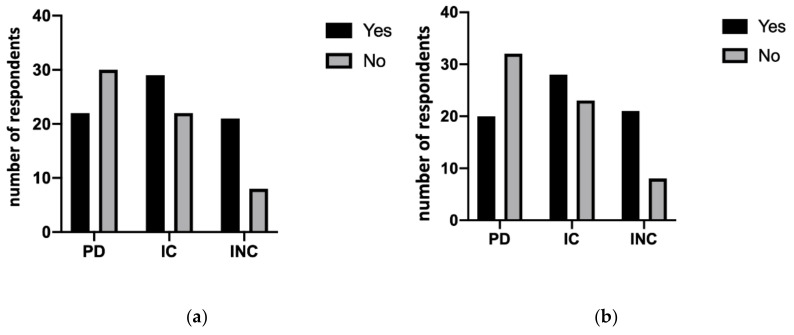
(**a**) The difficulty expressed by dentists to understand the needs of people with disabilities showed significant differences (*p* < 0.05) between the three groups. (**b**) It also showed the necessity of dentist to stop any treatment (yes) or not (no) (*p* < 0.05).

**Figure 4:**
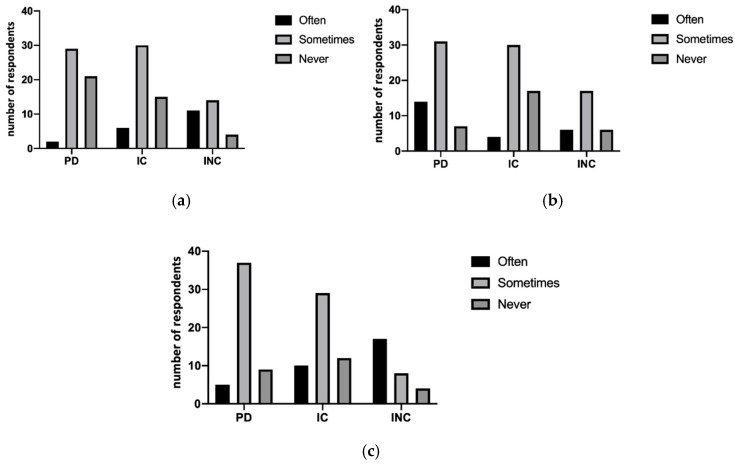
The graphics represent the difference between the groups in the need of special treatments: necessity of sedation use (*p* < 0.01) (**a**), wheelchair (*p* < 0.05) (**b**) or special maintenance support (*p* < 0.01) (**c**) showed a statistically significant difference between groups.

Dentists also believe they have difficulty during the treatment of people with physical disabilities (42.3%), collaborating people with cognitive disabilities (56.9%) and non-collaborating people with cognitive disabilities (72.4%), with significant differences (*p* < 0.05) between the groups, as demonstrated in Figure 3a. Moreover, the occurrence of giving up treatment was shown in Figure 3b (*p* < 0.05). In this regard, the most frequent treatments are oral hygiene, extractions and first visit in more than 80% of the questionnaires analyzed. Other information involves the possibility of inclusion in follow-up programs of these people. The data report that over 50% of dentists who follow non-collaborating people with disabilities never or almost never plan to include them in follow-up programs. However, no statistically significant difference was shown between the treatment performed in different types of disabilities. On the contrary, statistically significant differences were shown between the strategy used in different types of disability with the use of sedation (*p* < 0.001), treatment of wheelchair (*p* < 0.05) and other specific protective supports (*p* < 0.001), as shown in Figure 4a–c.

With regard to the third and last section of the questionnaire, 76 (83.5%) of the interviewees suggested the presence of a specialist dentist trained for these kinds of treatments. On the other hand, 84 (92.3%) of dentists believe that dental disease is underestimated in people with disabilities, and 65.9% believe that the public and private dental offices dedicated to the treatment of these people are insufficient.

### 3.2. Results from People with Disabilities

With regard to the questionnaires issued to the ANFFAS population (both people with disabilities and their caregivers/family members), various important information emerged, as shown in Table 2. Out of the 61 respondents, 39.35% were male and 60.65% were women. Only one person reported to live alone. Only 9.83% of these live in a dedicated care center; meanwhile, the remaining people live with parents or family members. With regard to dental care, 30 (49.1%) people with disabilities reported that they never go to the dentist. Nevertheless, 28 (45.9%) declared to have gone to the dentist for the first time during childhood. Another piece of information that emerged is that 29 (47.54%) declared that they have many difficulties in finding a specialized dentist able to treat them. Moreover, 37 (60%) of the people with disabilities reported going to the dentist only when needed and mainly for professional oral hygiene, caries and extractions. Specifically, 28 (45.90%) go to the dentist for a control visit, 25 (40.98%) to make teeth extractions and 30 (49.18%) do not count professional oral hygiene among the services performed by dentists.

Of the respondents, 19 (31.14%) stated that they do not find staff who can understand their problems or have the tools to operate on them. In addition, 14 (22.95%) of them go to the dental department of the hospitals. Regarding the perceived dental health status, 13 (21.31%) reported having frequent gingival bleeding and 21 (34.42%) to have it only sometimes; 11 (18.08%) to have extracted many teeth and 31 (50.82%) only some teeth. A total of 12 (19.67%) know they have dental caries. Interestingly, 24 (39.34%) respondents declared that they need special help or assistance during treatment for myriad reasons and due to the individual needs of these people. Among these, they reported: special supports, difficulty in lying position, need for sedation or anesthesia and reduced chairside time. Only 12 (20%) people with disabilities or caregivers reported that they were included in follow-up programs.

## 4. Discussion

As described, the survey administration was made through paper and Google Form platform questionnaires. The dentists (91 persons) who completed the questionnaires had an average age of 33.9 years. Young dentists probably have less experience in the field but, at the same time, a greater sensitivity and interest was shown. Although the response rate was only 16.5%, it was sufficient to obtain the expected results and analyze the data based on the sample size calculation. These data could be read, as often happens in surveys, as part of the population to which the questionnaire is addressed is not interested, or has no time available. Furthermore, the electronic administration can discourage some users [25,26]. The null hypothesis in this study was rejected because differences were shown between the treatment and difficulties found in people with disabilities.

Only a small number of dentists reported that they easily treat patients with disabilities. By comparing these findings to the number of people with disabilities in the Abruzzo region (74,568 people reported in 2015), it is clear that we are still far from guaranteeing a valid service to this part of the population [2]. Some authors declare that the lack of patient autonomy may explain the poor request to access to dental care [27]

Related to dental treatments, 73.1% of dentists believe that people with physical disabilities do not have a good standard of oral hygiene. Over 80% of people with disabilities undergo exclusively defined emergency or primary necessity treatments. Conservative therapies, such as endodontic performances and reconstructions, are still scarce. Meanwhile, a high number of extracted dental elements in a population already in a disadvantaged situation was described. In the same way, prosthetic dental services, such as crowns, removable prostheses and implant supported restorations, are not very common among these patients, compared to the general dental panorama [28]. The same applies to the orthodontic field where our data demonstrated an almost complete absence of this kind of treatment. Indeed, the authors think that the latter, in a population where dental malposition and occlusal pathologies are very frequent [29,30], orthodontic treatment is almost necessary. Among predominant disorders, oral diseases are globally recognized and have serious health, social and economic implications, which significantly reduce the quality of life of people with disabilities [31]. Even for patients and caregivers, these data are extremely high. There are 60% who go to the dentist only in cases of necessity. The situation does not change even at an international level where the aspect linked to the follow-up of people with disabilities is scarcely taken into consideration [32]. The Italian ministerial guidelines suggest that patients should be included in a personalized follow-up program [22,23]. In addition, over 50% of dentists who follow non-collaborating patients never or almost never plan to include them in follow-up programs. These data are particularly shocking as hygienic maintenance and follow-up are essential strategies for maintaining the good health of patients [33]. In the authors’ opinion, it is likely that the management software and use of dedicated staff could help dentists and dental hygienists in the periodic control of patients, possibly by dedicating specific days and more treatment time for their needs. Data collected through a survey conducted by the Italian Society of Periodontology (SIdP) showed a complex panorama with high tooth mortality in the general population, underlining the importance of undertaking public health policies aimed at promoting oral health to reduce tooth loss and the onset of related systemic diseases [34].

On the other hand, the present data showed that although problems emerged, linked to the presence of architectural barriers or equipment (dental chair) not comfortable and suitable for these people, patients with physical disabilities will be more easily accepted into the dental practice in the Abruzzo region than those with cognitive impairments. Other studies report that only a third of dentists have a suitable accommodation facility, effectively restricting access to a large portion of the population [12]. Moreover, in line with national guidelines, our data show that in the Abruzzo region the problem of removing architectural barriers has been recognized by the dentists who want to treat these kinds of patients.

A total of 80.2% of dentists agree with attending courses or training days. A study conducted in Greece showed that less than 40% of dentists received adequate training to treat people with disabilities [6]. Similar results were reported in a Belgian study [35]. Based on the results, the treatment of these patients is critical, which is not surprising and in line with the international literature [36,37]. However, other authors have stressed the request raised by clinicians for specialised training, as well as the lack of adequate training itself [38,39]. From the patient’s point of view, difficulties during treatment emerge, such as the need for particular assistance or sedation and the difficulty to lie down in the dentist’s chair. Clearly, the objective of this survey was to provide a regional picture of the problem. Comparing these results with an international panorama aims to highlight critical or more positive aspects that emerged from this survey. This allows us to recognize the aspects that require the most improvement with respect to international situations investigated with the same methodology. However, the sensitivity towards social issues and the care of socially disadvantaged people should be a priority universally in the world. The critical issues that emerge should be considered wherever you are in the world. Improving the conditions of these patients remains a common goal.

The data presented here are an excellent starting point for improving the approach and treatment of patients with disabilities. The literature has already demonstrated that surveys are useful to better understand various problems and evaluate the appropriate solutions [40,41].

The information gathered from dentists and patients can provide concrete assistance to take care of oral health and complete some of the challenges faced by these two populations, improving dental treatments and being able to better serve patients with disabilities. Despite the enormous efforts made by hospitals and the Ministry to regulate such dental care, the number of patients who benefit from it is still small. Specialized training courses, inclusion in follow-up programs, use of dedicated equipment and facilities can increase the quality and access to care for this part of the population. Although the reported results are very interesting, there are different limitations in the present study. First of all, it reports the results of a regional survey, which makes it mandatory to further investigate this subject on a national or international scale. Moreover, a larger sample could be important to obtain more generalizable data. Furthermore, the survey does not allow for a temporal relationship between exposure and outcome. Thus, the conclusions should be taken into consideration as part of the survey in a local area. Despite the easy-to-read language adopted for the questionnaire of the present study, it will be important to define other types of questionnaires suitable for all kinds of people with disabilities in order to better understand their needs. In the presented data, if the patient was unable to answer the questions, the questionnaire was submitted to their caregiver or their family members who live with them and hopefully are familiar with their moods and difficulties. However, this may be an inaccurate assumption and might have been a bias in our study. In addition, an extension of the survey to other medical fields could help to further investigate the treatment of these patients and how this can be improved. Further studies could be carried out to practically evaluate the effectiveness of follow-up programs in these categories of patients to evaluate their long-term effects in maintaining oral health.

## 5. Conclusions

It can be assumed that a reduced amount of people with disabilities have access to the dental care. In addition, people with disabilities report a difficulty in finding specialized dentists and barrier-free dental offices. Thus, greater attention to the requests of people with disabilities (barrier-free dental offices, specialized dentists and dental offices equipped with the necessary tools) and effective communication between themselves and dentists may improve their oral health condition. Moreover, inclusion in follow-up programs could facilitate the management of dental problems, which would then avoid having to manage almost solely emergency therapies and reduce the gap between these people and the rest of the population. Moreover, specialized dental training courses should also be increased to offer a wide range of options to these people. Finally, further investigation is necessary to better understand the needs of people with disabilities, which could be the best way to increase their oral health conditions. We, therefore, hope that our reflections can help researchers and dentists to optimize the care of patients with disabilities, reflecting on the need for follow-up or on the differentiated approach to such patients.

## Figures and Tables

**Table 1:** Summary table of demographic data and main results obtained by administering questionnaires to dentists. (*) Good oral hygiene was established by the common index used daily for patient monitoring.

Demographic Data			
Number of participants	91		
Mean age	33.90 y		
Sex (male)	60.4% (*n* = 55)		
Post graduate education (yes)	38.5% (*n* = 35)		
Treat people with disabilities (yes)	69.2% (*n* = 63)		
**Main Results**	Physical disability	Collaborating cognitive disabilities	Non-collaborating cognitive disabilities
Percentage of patient treated	82.5%	81%	46%
Patient treated per year (<10/per year)	73.5%	76.5%	93%
Specific training of the dental team (yes)	32.7% (35)	33.3% (17)	41.4% (12)
Good (*) oral hygiene (yes)	26.9% (14)	15.7% (8)	13.8% (4)
Difficulty in interaction (yes)	42.3% (23)	56.9% (29)	72.4% (21)
*Main Difficulties*			
Presence of architectural barriers in the structure	52.2%	22.6%	18.2%
Difficulty in moving the patient with disabilities	56.5%	19.4%	31.8%
Difficulty interacting with the patient with disabilities	73.9%	90.3%	90.9%
Lack of suitable equipment	8.7%	25.8%	36.4%
Need to use coercive methods	13%	29%	40.9%
Lack of time to carry out the treatments	17.4%	32.3%	27.3%
Having difficulty in finishing dental care (yes)	38.5%	45.1%	72.4%
*Main Treatments Carried Out*			
Medical examination	82.7%	86.3%	86.2%
Oral hygiene	88.5%	86.3%	75.9%
Restorative dentistry	65.3%	60.8%	44.8%
Extractions	69.2%	60.8%	62.1%
Endodontics	42.3%	43.1%	20.7%
Dental implants	13.5%	11.8%	3.4%

**Table 2:** Summary table of demographic data and main results obtained by administering questionnaires to people with disabilities and/or their caregiver.

Demographic Data of 61 Participants	
People with disabilities who answered by themselves	35
Mean age (people with disabilities)	46.3 year
Sex (male) (people with disabilities)	42.85%
Caregivers answering on behalf of people with disabilities	26
Mean age (caregiver)	57.31 y
Sex (male) (caregiver)	34.61%
**Main Results**	
Was it easy to find a dentist? (yes)	52.46%
Do you go (or does the person who you assist) go to the dentist only when needed? (yes)	60%
Do you (or does the person who you assist) go to an hospital? (yes)	22.95%
Do your (or does the person’s who you assist) gums bleed? (yes)	21.31%
Do you (or does the person who you assist) extracted some teeth? (yes)	50.82%
Do you (or does the person who you assist) need special help during treatment? (yes)	39.34%
Can you (or the person who you assist) communicate your dental problems to the dentist? (yes)	68.86%
*Main Treatments Carried Out*	
Medical examination	45.9%
Oral hygiene	50.82%
Restorative dentistry	23.5%
Extractions	47.8%
Endodontics	32.3%
Dental implants	13.5%

## Supplementary Materials

The following are available online at https://www.mdpi.com/1660-4601/18/4/1556/s1, File S1: The informed consent form and the questionnaires.
