# Supplementary material for: Access to Dental Care—A Survey from Dentists, People with Disabilities and Caregivers
Source: Int J Environ Res Public Health. 2021 Feb 6;18(4):1556. doi: 10.3390/ijerph18041556 (PMC7915372; doi:10.3390/ijerph18041556)
Supplement: Supplementary file 1 [file ijerph-18-01556-s001.pdf]

INFORMATIVA SUL TRATTAMENTO DEI DATI PERSONALI

PER FINALITÀ DI RICERCA SCIENTIFICA

(ART. 13 REG. UE 2016/679)

Titolo del Progetto di ricerca: Barrier-free dentistry: the approach and treatment of patients with disabilities

---

Gentile partecipante,
desideriamo informarLa che la normativa vigente in materia di protezione dei dati, con particolare riguardo all'ambito della ricerca (Regolamento UE 2016/679 sulla protezione dei dati personali - GDPR, il D. lgs. n. 196/2003 "Codice in materia di protezione dei dati personali" come modificato dal D. lgs. 101/2018, le "Regole deontologiche per i trattamenti a fini statistici o di ricerca scientifica" - Provvedimento del Garante per la protezione dei dati personali n. 515 del 19 dicembre 2018 nonché le varie Prescrizioni del Garante in materia) sancisce il diritto di ogni persona alla protezione dei dati di carattere personale che la riguardano.

In conformità alla normativa citata il trattamento dei Suoi dati personali nell'ambito del progetto di ricerca sarà improntato al rispetto dei principi di cui all'art. 5 del GDPR e, in particolare, liceità, correttezza, trasparenza, pertinenza, non eccedenza ed in modo da garantire un'adeguata sicurezza dei dati stessi.

In qualità di Interessato, Le forniamo le seguenti informazioni relative al trattamento dei Suoi dati personali.

TITOLARE DEL TRATTAMENTO E RESPONSABILE DELLA PROTEZIONE DEI DATI

Il Titolare del trattamento è l'Università "G. d'Annunzio", Via Vestini 31, 66100 Chieti, email: ateneo@pec.unich.it.
Il Responsabile della protezione dei dati (RDP) di Ateneo è il Prof. Gianluca Bellomo e può essere contattato al seguente indirizzo email: dpo@unich.it.

FINALITÀ DEL TRATTAMENTO

Il trattamento dei Suoi dati personali è effettuato per la realizzazione delle finalità scientifiche del Progetto "Barrier-free dentistry: the approach and treatment of patients with disabilities"
Il Progetto è stato redatto conformemente agli standard metodologici del settore disciplinare interessato ed è depositato presso il Dipartimento di Scienze Mediche, Orali e Biotecnologiche dell'Università G. d'Annunzio Chieti-Pescara ove verrà conservato per due anni dalla conclusione programmata della ricerca stessa.

CATEGORIE DI DATI PERSONALI

Nella realizzazione del Progetto verranno trattati i seguenti dati personali: età, genere, provincia di lavoro e titolo di studio del partecipante.

BASE GIURIDICA DEL TRATTAMENTO

Il trattamento dei Suoi dati personali viene effettuato dal Titolare nell'ambito di esecuzione dei propri compiti di interesse pubblico (per finalità di ricerca scientifica) ai sensi dell'art. 6, par. 1, lett. e) del GDPR.

MODALITÀ DEL TRATTAMENTO

I Suoi dati personali saranno trattati esclusivamente da soggetti autorizzati nell'ambito della realizzazione del Progetto.

Il trattamento dei Suoi dati personali è effettuato, per mezzo delle operazioni o complesso di operazioni indicate dalla sopra richiamata definizione normativa di "trattamento", con l'ausilio di strumenti elettronici.

PERIODO DI CONSERVAZIONE DEI DATI

I Suoi dati personali saranno conservati presso il Settore Progetti Nazionali e Europei, in uno specifico fascicolo di progetto, sia cartaceo che informatico, per un massimo di due anni; entro i due anni successivi alla presentazione del progetto saranno trasferiti, unitamente al relativo fascicolo di progetto, all'archivio di deposito dell'Ateneo

NATURA DEL CONFERIMENTO DEI DATI

Il conferimento dei Suoi dati per le suddette finalità di ricerca è indispensabile per lo svolgimento del Progetto ed il mancato conferimento determina l'impossibilità di parteciparvi.

DIVULGAZIONE DEI RISULTATI DELLA RICERCA

La divulgazione dei risultati statistici e/o scientifici (ad esempio mediante pubblicazione di articoli scientifici e/o la creazione di banche dati, anche con modalità ad accesso aperto, partecipazione a convegni, ecc.) potrà avvenire soltanto in forma anonima e/o aggregata e comunque secondo modalità che non La rendano identificabile.

DIRITTI DELL'INTERESSATO

In qualità di Interessato ha diritto di chiedere in ogni momento al Titolare l'esercizio di diritti di cui agli artt. 15 e ss. del GDPR e, in particolare, l'accesso ai propri dati personali, la rettifica, l'integrazione, nonché se ricorrono i presupposti normativi, la cancellazione degli stessi, la limitazione del trattamento dei dati e il diritto di opporsi al loro trattamento. Per l'esercizio dei diritti e per informazioni relative al Progetto può rivolgersi al Responsabile scientifico del Progetto al seguente recapito: b.sinjari@unich.it. Resta salvo il diritto di proporre reclamo all'Autorità Garante per la protezione dei dati personali ai sensi dell'art. 77 del GDPR.

Questionnaire for the dentist

Instructions: Sign with an "X" to answer the following questions:

I consent, as a doctor or dentist, to the processing of data and to the collection of information anonymously and for scientific purposes (based on art. 13 of Legislative Decree 196/2003 and art. 13 GDPR 679/16.)

1. Age: ________ Sex: M F

2. Please indicate the province / provinces in which you operate (even more than one answer):

Chieti____

Pescara___

Teramo___

L'Aquila ___

3. You have a degree in:

Medicine and Surgery ___

Dentistry ___

4. Do you have a post graduate education?

Yes ___

No___

5. If yes, Which one of this?

Oral surgery ____

Orthodontics ____

Paediatric dentistry ____

Clinical-general dentistry ____

Maxillofacial surgery ____

Other (__________________)

6. Are you aware of the legislation for the treatment of people with disabilities?

Yes___

No ___

7. Where do you work?

Private practice ____

University Dental Clinic ____

Hospital____

Other (_____________________)

8. Do you know a branch of dentistry called Special Dentistry?

Yes____

No____

9. Do you treat / have you ever treated people with disabilities?

Yes____

No____

The questions in the next three sections are only for those who answered “Yes” to question 9.

If you answered “no”, you can continue to answer from question 56

10. Which people with disabilities do you treat? (Answer each category with Yes or No):

People with physical disabilities ___

People collaborating with cognitive disabilities ___

People non-collaborating with cognitive disabilities ___

Questions related to the treatment of people with physical disabilities:

11. How many people with physical disabilities do you treat in one year?

Less than 10___

Between 10 to 20____

Between 21 to 30____

More than 30___

12. How many percentages of people with physical disabilities do you treat out of the total number of people?

Less than 20% ___

Between 20% and 50% ___

More than 50% ___

13. Have you participated in specific training for the examination and treatment of people with physical disabilities?

Yes ____

No ____

14. Do you carry out home visits for the visit and care of people with physical disabilities?

Yes ____

No ____

15. Do the patients you visit with physical disabilities have proper oral hygiene?

Yes ___

No___

16. (If you answered “no” to the previous question) Why? (even more than one answer)

Lack of dedicated tools____

Lack of coordination in movements ____

Lack of oral health care____

Lack of time to carry out the treatments ____

Lack of information or knowledge of the topic ____

Other (_________________________________)

17. Do you provide correct oral hygiene instructions for patients with physical disabilities that you treat?

Yes ___

No___

18. (If you answered “yes” to the previous question) Who do you provide the correct oral hygiene instructions to (even more than one answer)?

To the person with disabilities ___

To the parents of the person with disabilities ___

To the spouse of the person with disabilities ___

To the child of the person with disabilities ___

To the Caregiver of the person with disabilities___

Other (____________________)

19. Do you have difficulty interacting and / or treating people with physical disabilities?

Yes___

No___

20. (If you answered “yes” to the previous question) What are the main difficulties you have? (even more than one answer)

Presence of architectural barriers in the structure _____

Difficulty in moving people with disabilities _____

Difficulty interacting with people with disabilities _____

Lack of suitable equipment _____

Need to use coercive methods _____

Lack of time to carry out the treatments_____

Other (____)

21. Do you need special support (compared to conventional treatment) in treating people with physical disabilities? (Please reply with “often” ,“Sometimes” or “Never”)

Protection or maintenance support _______

Sedation _____

General anesthesia _____ ___________ _

Wheelchair intervention _____ ___________ _____

Special chair, backrest, headrest _____ ___________ _____

Other (____)

22. What kind of dental treatment do you perform?

Medical examination ___

Intraoral radiographs ___

Dental fluor prophylaxis ___

Tartar removal ___

Oral hygiene instructions ___

Fixed prosthesis ___

Restorative dentistry ___

Dental extractions ___

Dental groove sealing ___

Fixed and mobile orthodontic appliances ___

Partial or total removable prosthesis ___

Dental bleaching ___

Endodontics ___

Dental implants ___

Oral surgery ___

I don't know how to answer _____

Other (____)

23. Once the treatment plan is finished, does the patient with a disability enter a follow-up program?

Always ___

Often ___

Sometimes___

Never ___

I don’t know ___

24. Have you ever had difficulty finishing dental care for people with physical disabilities?

Yes ___

No ___

25. (If you answered “yes” to the previous question) why?

Questions related to the treatment of people collaborating with cognitive disabilities:

26. How many people collaborating with cognitive disabilities do you treat in one year?

Less than 10___

Between 10 to 20____

Between 21 to 30____

More than 30___

27. How many percentages of people collaborating with cognitive disabilities do you treat out of the total number of people?

Less than 20% ___

Between 20% and 50% ___

More than 50% ___

28. Have you participated in specific training for the examination and treatment of people collaborating with cognitive disabilities?

Yes ____

No ____

29. Do you carry out home visits for the visit and care of people collaborating with cognitive disabilities?

Yes ____

No ____

30. Do the patients collaborating with cognitive disabilities you visit have proper oral hygiene?

Yes ___

No ___

31. (If you answered “No” to the previous question) Why? (even more than one answer)

Lack of dedicated tools____

Lack of coordination in movements ____

Lack of oral health care____

Lack of time to carry out the treatments ____

Lack of information or knowledge of the topic

Other(___________________)

32. Do you provide correct oral hygiene instructions for people collaborating with cognitive disabilities?

Yes ___

No ___

33. (If you answered “yes” to the previous question) Who do you provide the correct oral hygiene instructions to (even more than one answer)?

To the person with disabilities ___

To the parents of the person with disabilities ___

To the spouse of the person with disabilities ___

To the child of the person with disabilities ___

To the Caregiver of the person with disabilities___

Other (_____________________)

34. Do you have difficulty interacting and / or treating people collaborating with cognitive disabilities?

Yes ___

No ___

35. (If you answered “yes” to the previous question) What are the main difficulties you have? (even more than one answer)

Presence of architectural barriers in the structure _____

Difficulty in moving people with disabilities _____

Difficulty interacting with people with disabilities _____

Lack of suitable equipment _____

Need to use coercive methods _____

Lack of time to carry out the treatments_____

Other (____)

36. Do you need special support (compared to conventional treatment) in treating people collaborating with cognitive disabilities? (Please reply with “often” ,“Sometimes” or “Never”)

Protection or maintenance support _______

Sedation _____

General anesthesia _____ ___________ _

Wheelchair intervention _____ ___________ _____

Special chair, backrest, headrest _____ ___________ _____

Other (____)

37. What kind of dental treatment do you perform?

Medical examination ___

Intraoral radiographs ___

Dental fluor prophylaxis ___

Tartar removal ___

Oral hygiene instructions ___

Fixed prosthesis ___

Restorative dentistry ___

Dental extractions ___

Dental groove sealing ___

Fixed and mobile orthodontic appliances ___

Partial or total removable prosthesis ___

Dental bleaching ___

Endodontics ___

Dental implants ___

Oral surgery ___

I don't know how to answer _____

Other (____)

38. Once the treatment plan is finished, does the patient collaborating with cognitive disability enter a follow-up program?

Always ___

Often ___

Sometimes___

Never ___

I don’t know ___

39. Have you ever had difficulty finishing dental care for people collaborating with cognitive disabilities?

Yes ___

No ___

40. (If you answered “yes” to the previous question) why?

Questions related to the treatment of people non-collaborating with cognitive disabilities:

41. How many people non-collaborating with cognitive disabilities do you treat in one year?

Less than 10___

Between 10 to 20____

Between 21 to 30____

More than 30___

42. How many percentages of people non-collaborating with cognitive disabilities do you treat out of the total number of people?

Less than 20% ___

Between 20% and 50% ___

More than 50% ___

43. Have you participated in specific training for the examination and treatment of people non-collaborating with cognitive disabilities?

Yes ____

No ____

44. Do you carry out home visits for the visit and care of people non-collaborating with cognitive disabilities?

Yes ____

No ____

45. Do the patients non-collaborating with cognitive disabilities you visit have proper oral hygiene?

Yes ___

No ___

46. (If you answered "No" to the previous question) Why? (even more than one answer)

Lack of dedicated tools____

Lack of coordination in movements ____

Lack of oral health care____

Lack of time to carry out the treatments ____

Lack of information or knowledge of the topic

Other(___________________)

47. Do you provide correct oral hygiene instructions for people non-collaborating with cognitive disabilities?

Yes ___

No ___

48. (If you answered "yes" to the previous question) Who do you provide the correct oral hygiene instructions to (even more than one answer)?

To the person with disabilities ___

To the parents of the person with disabilities ___

To the spouse of the person with disabilities ___

To the child of the person with disabilities ___

To the Caregiver of the person with disabilities___

Other (____________________)

49. Do you have difficulty interacting and / or treating people non-collaborating with cognitive disabilities?

Yes ___

No ___

50. (If you answered "yes" to the previous question) What are the main difficulties you have? (even more than one answer)

Presence of architectural barriers in the structure _____

Difficulty in moving people with disabilities _____

Difficulty interacting with people with disabilities _____

Lack of suitable equipment _____

Need to use coercive methods _____

Lack of time to carry out the treatments_____

Other (____)

51. Do you need special support (compared to conventional treatment) in treating people non-collaborating with cognitive disabilities? (Please reply with "often" ,"Sometimes" or "Never")

Protection or maintenance support _______

Sedation _____

General anesthesia _____ ___________ _

Wheelchair intervention _____ ___________ _____

Special chair, backrest, headrest _____ ___________ _____

Other (____)

52. What kind of dental treatment do you perform?

Medical examination ___

Intraoral radiographs ___

Dental fluor prophylaxis ___

Tartar removal ___

Oral hygiene instructions ___

Fixed prosthesis ___

Restorative dentistry ___

Dental extractions ___

Dental groove sealing ___

Fixed and mobile orthodontic appliances ___

Partial or total removable prosthesis ___

Dental bleaching ___

Endodontics ___

Dental implants ___

Oral surgery ___

I don't know how to answer ____

Other (____)

53. Once the treatment plan is finished, does the patient non-collaborating with cognitive disability enter a follow-up program?

Always ___

Often ___

Sometimes___

Never ___

I don't know ___

54. Have you ever had difficulty finishing dental care for people non-collaborating with cognitive disabilities?

Yes ___

No ___

55. (If you answered "yes" to the previous question) why?

Questions related to improving the treatment of patients with disabilities

56. Do you think the presence of a professional trained in special dentistry is necessary in the office?

Yes ___

No ___

57. What suggestions would you give to improve the approach to patients with disabilities?

____

58. Do you think that a specific preparation (besides the university one) is necessary to carry out the best dental treatment in patients with disabilities?

Yes ___

No ___

I do not know ____

59. Do you think dental disease is neglected by patients and caregivers of people with disabilities?

Yes ___

No ___

60. (If you answered “yes” to the previous question? Why? (Even more than one answer)

Economic reasons___

Cultural reasons ___

Lack of suitable facilities ___

Underestimation of dental pathology with respect to general problems ___

Other (____)

61. Are you aware of the possibility of performing oral diagnostic screening for patients with disabilities?

Yes ___

No ___

62. Do you think that the Essential Levels of Assistance (LEA) are sufficient for the prevention, diagnosis and treatment of dental diseases in people with disabilities?

Yes ___

No ___

I don’t know ___

63. Do you think that people with disabilities can be treated in private dental practices or in dedicated facilities?

Yes ___

No ___

I do not know ___

64. Are you interested in increasing training for the treatment of people with disabilities?

Yes ___

No ___

Questionnaire for the caregiver / family member

Instructions: Sign with an "X" to answer the following questions:

I consent to the processing of data and the collection of information anonymously and for scientific dissemination purposes based on art. 13 of Legislative Decree 196/2003 and art. 13 GDPR 679/16.

1. Age: ________ Sex: M ___ F____

2. Please indicate the city of residence: ____________________

3. Type of relationship with the person with disability:

Parent ___

Son ___

Spouse ___

Caregiver ___

Other: ____

4. Type of disability:

physical disability ___

cognitive collaborating disability ___

cognitive non-collaborating disability ___

5. Age of the person with disabilities: ____

6. Has the person with disabilities ever had dental problems ?

Often___

Sometimes ___

Never ___

7. Does the person with disabilities have difficulty communicating problems with the dentist?

YES___

NO___

8. Can the person with disabilities communicate dental problems or pains to you?

Yes ___

No ___

9. If you answered “no”, Do you independently identify the changes in behavior and / or mood of the person with disability due to dental problems, in order to be able to carry out treatments immediately?

Always___

Sometimes ___

Never ___

10. Does the person with disabilities go to the dentist independently?

Always ___

Sometimes __

Never ___

11. At what age did the person with disabilities go to the dentist for the first time?

Childhood ___

Adolescence ___

Adult ___

I don’t know ___

12. Has the person with disabilities found it difficult to find a dentist who can treat him properly?

No, It was easy ___

Yes, It was necessary to evaluate different professionals ___

Yes, It hasn't been found yet ___

13. When was the last dental visit?

Less than six months ___

Between six months and a year ___

More than a year ___

I don’t remember ___

14. What kind of dental treatment do you received?

Medical examination ___

Intraoral radiographs ___

Dental fluor prophylaxis ___

Tartar removal ___

Oral hygiene instructions ___

Fixed prosthesis ___

Restorative dentistry ___

Dental extractions ___

Dental groove sealing ___

Fixed and mobile orthodontic appliances ___

Partial or total removable prosthesis ___

Dental bleaching ___

Endodontics ___

Dental implants ___

Oral surgery ___

I don't know how to answer _____

Other (____)

15. How often does the person with disability go to the dentist?

Only for emergency ___

Every 6 months ___

At least once a year ___

Other ____________

16. How far is the dentist from the place of residence?

Less than 10 km ___

Between 10 and 50 km ___

Over 50 km ___

17. The reference dental practice is:

A private dental practice ___

An Hospital ___

A university clinic ___

Other (_____________________)

18. Does the person with disabilities need special support compared to conventional treatment?

No ___

Protection or maintenance support ___

Sedation ___

General anesthesia ___

Wheelchair intervention ___

Special chair, backrest, headrest ___

Other (_______________)

19. Has the person with disability ever left the dental session without finishing the treatment?

Often ___

Sometimes ___

Never ___

20. Have you ever asked for a home visit due to the inability to go to the dental office?

Yes ___

No ___

21. (If you answered “YES” to the previous question) Did you get such visits?

Yes ___

No ___

22. If the person with disability has difficulty accessing dental care, he can specify the reasons (Reply with a “Often”, “Sometimes” or “Never”)

-In the study there are no adequate spaces and equipment: ____

-The dentist does not have adequate training to treat person with disabilities: ____

-The dentist does not want to carry out the dental treatment:____

-Economic reasons: ____

-Difficulty sitting in the dentist's chair: _____

-Difficulty in transport or to reach the structure: ____

-Fear of the dentist: ____

-Lack of time: ___

- Due to major health problems: ____

23. Is dental health important for the general health of the person with disabilities?

Very important ____

Quite important ____

Not very important ____

Not at all important ____

24. Do you think that the pathology or the drugs that person with disabilities take are responsible for the worsening of the dental situation?

Yes ___

No ___

I don't know ___

25. Has the dentist who takes care of the patient with disability included him in a follow-up program?

Yes ___

No ___

I do not know ___

26. Do you think that the dentist is prepared to treat people with disabilities?

Yes ___

No ___

I don’t know ___

27. Is the person with disabilities able to independently take care of his oral hygiene?

Yes ___

NO ___

28. (If you answer “No” to the previous question) Who takes care of oral hygiene?

One of the family members ___

More family members / facility ___

I don't know how to answer ___

29. What are the main difficulties that people with disabilities have during oral hygiene?

Difficulty keeping the toothbrush in the correct position ___

Difficulty in making the correct movement of the toothbrush ___

Difficulty knowing if he has cleaned his teeth properly ___

Other (____________________________)

30. How often does the person with disability (or who is taking care) brush their teeth?

Once a day___

Twice daily___

Three times a day___

Other________

31. What does the patient with a disability (or caregiver) use for oral hygiene?

Manual toothbrush ___

Electric toothbrush ___

Dental floss ___

Mouthwash ___

Other ________________________________

32. Does the person with disabilities have many cavities?

Yes ___

NO ___

I don’t know ___

33. Does the person with disabilities have gingivitis problems?

Never ___

Sometimes ___

Often ___

I don’t know ___

34. Has the person with disabilities had dental extractions?

No ___

Some teeth ___

Many teeth ___

I don’t know ___

35. Do you think the person with disabilities has bruxism problems?

Yes ___

NO ___

I don’t know ___

Questionnaire for the patient with disabilities

Before asking you questions about the dentist, we ask you if we can read and use the answers you give us right now to do scientific research.

There is a legislative decree, which is a type of law made by the state, with a European regulation that in article 13 say that we can use your answers for our research.

This legislative decree is the decree number 196/2003 and is called the personal data protection code.

The European regulation is the number 679/16 and is called regulation regarding the protection of individuals with regard to the processing of personal data and the free movement of such data.

Your answers, however, are anonymous and this means that we don't tell anyone that you are the one who gives us these answers. We don't tell your name and surname to anyone. At the end of these questions you don't need to put your signature on the document.

The questions we ask you and which we ask you to answer concern the dentist. We ask you to answer by writing your data, for example how old you are and where you live, or to put an X sign in the square next to the answer that seems most right for you

Instructions: Sign with an "X" to answer the following questions:

❑ consent, as a directly interested subject, to the processing of data and the collection of information anonymously for scientific purposes based on art. 13 of Legislative Decree 196/2003 and art. 13 GDPR 679/16.

1. **How old are you?** _________
2. **Are you a male or a female?** _________
3. **Where do you live?** _________
4. **Who do you live with?**

   With my family _________

   Alone _________

   In a facility with other people _________
5. **Have you ever gone to the dentist for dental problems?**

   Often _________

   Sometimes _________

   Never _________

6. **How old were you when you first went to the dentist?**

   When I was a child _________

when I was an adolescent ________

when I was an adult ________

I don’t remember ________

7. **Was it easy to find a dentist to treat your teeth?**

Yes, I found it easily ________

No, I changed several dentists ________

No, I haven't found a dentist yet ________

8. **When was the last time you went to the dentist?**

Less than six months (not long ago) ________

Between six months and a year (last year) ________

More than a year ago (write the date) ________

I do not remember ________

9. **What did you do to the dentist?**

I made a Visit ________

I made x-rays, which are photos of my teeth ________

I brush my teeth ________

I put a fixed prosthesis, i.e. a capsule or crown that covers the tooth ________

I treated caries ________

I removed some teeth ________

I put braces to make my teeth straighter ________

I put the denture ________

I whitened my teeth, that is I made them turn whiter ________

I did the root canal therapy , in fact they removed the nerve that is inside the tooth

I placed implants in the teeth, such as a screw in the tooth ________

I can not answer ________

10. How often do you go to the dentist?

Only if a tooth hurts ________

Every 6 months ________

Once a year ________

Other, i.e. none of the answers is fine ________

11. Is the dentist near the place where you live?

It is very close, about 10 kilometres from home ________

Not very far, between 10 and 50 kilometres from home ________

it's more than 50 kilometres away from home ________

12. Where does your dentist work?

In a private office, that is, in a place which is not in a hospital _______

in a hospital ________

in a university dental clinic, that is, in a hospital that it's also a university where only teeth are cared for, in fact university means that the doctors who work they are also professors who teach at university

____

Other, that is, if none of the answers are correct, ________

13. Can you explain to the dentist what kind of dental problem you have, such as which tooth hurts you?

Yes ________

No ________

14. Do you need special assistance when treating your teeth?

No ________

I need help to lie down in the dentist's chair ________

I need to be sedated to relax, in fact I need to be relaxed with some medicine _______

I need general anesthesia, in fact I have to be asleep so that my teeth can heal ________

I need to brush my teeth in the wheelchair because I can't move ________

I need to brush my teeth on a special chair, for example with a backrest or headrest ________

Other, that is, if none of the answers are correct, write what kind of assistance do you need _____

15. Have you ever walked away from the dentist without having finished caring for your teeth? For example, were they treating you with tooth decay and you wanted to leave before they finished treating you?

Never ________

Sometimes ________

Often ________

16. **Have you ever asked the dentist to visit you at home or anywhere other than his or her dental office?**

Yes ________

No ________

17. If you answered yes to question 16, did the dentist visit you anywhere other than his or her dental office?

Yes ________

No ________

18. Do you have difficulties on treating your teeth at the dentist? (answer by putting an x to say if you often have difficulty, sometimes you have difficulty or you never have difficulty)

	Often	Sometimes	Never
Do you have difficulties because in the office there are no spaces or tools suitable for you?			
Are you having difficulties because the dentist was not able to cure you?			
Are you having difficulties because the dentist tells you that he cannot treat people with disabilities?			
Do you have difficulties because you have economic problems, or money problems?			
Do you have difficulties because you can't lie on the dentist's chair?			
do you have difficulties on going to the dentist's office?			

Do you have difficulties because you are afraid of the dentist?			
Do you have difficulties because you don't have time?			
Do you have difficulties because you have other health problems that need to be treated?			

19. Do you think that the care of your teeth is important for your health?

Very important ________

Quite important ________

Not very important ________

Not at all important ________

20. Can you brush your teeth yourself?

Yes ________

No ________

21. If you answered NO to question number 20, what difficulties do you have when brushing your teeth?

I can't keep the toothbrush the right way ________

I move the toothbrush the wrong way ________

I don't understand if I brushed my teeth well ________

Other, that is, if none of the answers are correct, you write what difficulty you have

____

22. If you answered no to question number 20, who helps you brush your teeth?

One of my family members ________

More people within my family / facility ________

I can not answer ________

23. How many times a day do you brush your teeth?

Once a day ________

Twice daily ________

Three times a day ________

Other, that is, if none of the answers are correct; You write how many times you brush your teeth in one day ________

24. What do you use to brush your teeth?(you can put multiple crosses on this question)

Manual toothbrush ________

Electric toothbrush ________

Dental floss ________

Mouthwash ________

Other, if none of the answers are correct: Write what you use to brush your teeth _______

25. Do you think you have a lot of tooth decay?

Yes ________

No ________

I do not know ________

26. Do your gums bleed, ie do you see blood when you brush your teeth or do you feel a strange taste of blood in your mouth?

Never ________

Sometimes ________

Often ________

I do not know ________

27. Did you remove many teeth?

No, none ________

Yes, Someone ________

Yes, many ________

I do not know ________

28. Are you thinking of tighten your teeth when you sleep, or while moving your teeth here and there with your mouth closed?

Yes ________

No ________

I do not know ________

Thanks for completing this questionnaire
